# The Plant Invader *Alternanthera philoxeroides* Benefits from Clonal Integration More than Its Native Co-Genus in Response to Patch Contrast

**DOI:** 10.3390/plants12122371

**Published:** 2023-06-19

**Authors:** Wenhua You, Ningning Li, Jin Zhang, Ao Song, Daolin Du

**Affiliations:** 1 Institute of the Environment and Ecology, College of the Environment and Safety Engineering, Jiangsu University, Zhenjiang 212013, China; 2 Jiangsu Collaborative Innovation Center of Technology and Material of Water Treatment, Suzhou University of Science and Technology, Suzhou 215009, China

**Keywords:** plant invasion, *Alternanthera philoxeroides*, heterogeneity, physiological integration, *Alternanthera sessilis*

## Abstract

Different connected parts of clonal plants often grow in different patches and the resource contrast between patches has an important effect on the material transfer between the connected ramets. However, it is unclear whether the effect of clonal integration differs between the invasive clonal plant and the related native species in response to patch contrast. To explore this, we grew the clonal fragment pairs of plant invader *Alternanthera philoxeroides* and its co-genus native species *A. sessilis* under high contrast, low contrast, and no contrast (control) nutrient patch environments, respectively, and with stolon connections either severed or kept intact. The results showed that, at the ramet level, clonal integration (stolon connection) significantly improved the growth of apical ramets of both species, and such positive effects were significantly greater in *A. philoxeroides* than in *A. sessilis*. Moreover, clonal integration greatly increased the chlorophyll content index of apical ramets and the growth of basal ramets in *A. philoxeroides* but not in *A. sessilis* under low and high contrast. At the whole fragment level, the benefits of clonal integration increased with increasing patch contrast, and such a positive effect was more pronounced in *A. philoxeroides* than in *A. sessilis*. This study demonstrated that *A. philoxeroides* possesses a stronger ability of clonal integration than *A. sessilis*, especially in patchy environments with a higher degree of heterogeneity, suggesting that clonal integration may give some invasive clonal plants a competitive advantage over native species, thus facilitating their invasion in patchy habitats.

## 1. Introduction

Biological invasions can reduce native species diversity and result in negative economic and human health impacts worldwide [1,2,3]. Numerous studies have been conducted to identify and understand the mechanisms underlying the process of invasion, and some inherent traits associated with clonal growth are widely recognized as critical determinants contributing to plant invasiveness [4,5]. Most notably, many of the most notorious invasive exotic plants are clonal plants [6,7,8], such as *Alternanthera philoxeroides* (Mart.) Griseb., *Solidago canadensis* L., and *Eichhornia crassipes* (Mart.) Solms. This raises the question of whether clonal traits exist in some invasive clonal plant species that give them an advantage over native clonal plants or non-clonal plants [9].

A unique feature of clonal plants is clonal integration, i.e., the transfer of water, nutrients, and photosynthetic products through vascular bundles driven by source–sink relationships that exist between clonal ramets [10,11]. The resources (e.g., light, water, and nutrients) required for plant growth and reproduction and the environmental conditions (e.g., temperature, humidity, disturbance, foraging) under which they live often exhibit heterogeneous distribution patterns [12,13]; thus, the physically interconnected ramets of a clonal plant with a strong capacity for horizontal expansion can often experience different resource and environmental patches [14,15]. Many studies have shown that in heterogeneous habitats, ramets growing in resource-rich patches can support the growth of ramets in resource-poor patches through clonal integration and thus improve the fitness of the whole clone [4,5,16]. Therefore, clonal integration is considered to be an important mechanism for the adaptation of clonal plants to the prevailing heterogeneity [17,18,19]. 

A recent focus of research is to compare differences in the ability of clonal integration between invasive alien species and native species. You et al. (2014) found that, relative to the native co-existing species *Jussiaea repens*, the invasive species *A. philoxeroides* exhibited greater clonal integration under heterogeneous resources and competitive environmental conditions, resulting in greater competitive ability and growth performance. The experimental results of Wang et al. (2017) also showed that invasive species benefited more from clonal integration than native species, leading to invasive species producing more biomass than native species. Therefore, clonal integration may confer a competitive advantage to invasive species relative to native species [5]. 

Contrast, which refers to the extent of the relative variation in resource availability between patches or between a patch and its surrounding matrix, constitutes one aspect of environmental heterogeneity [20]. Although environmental heterogeneity is universal, the contrast of heterogeneous patches is not the same [21], and the magnitude of contrast has a decisive influence on the transport of material between connected plant ramets [20]. The benefits of clonal integration may increase with increasing resource contrast between patches of heterogeneous environments [22,23]. However, experimental evidence on the relationship between patch contrast and clonal integration remains scarce [24,25]. In addition, it is still unclear whether this pattern differs between invasive clonal plant species and native clonal plant species. More profoundly, it means that patch contrast may affect differences in the ability of clonal integration between invasive clonal species and native clonal species, which is particularly important for our understanding of the relationship between clonal traits and plant invasion. Unfortunately, very few studies have tested this.

Through a controlled greenhouse experiment, we conducted a comparative analysis between the notorious invasive plant *A. philoxeroides* and its related native counterpart, *A. sessilis*. To investigate the effects of clonal integration on the growth performance of these two clonal plants in response to patch contrast, we grew the clonal fragments of both species under high, low, or no contrast of soil nutrients, respectively, and with stolon connections either severed (clonal integration prevented) or kept intact (clonal integration allowed). Based on past studies, we expect that increasing the contrast of resource patches will increase the benefits of clonal integration and that this pattern will be more pronounced in *A. philoxeroides.*

## 2. Results

### 2.1. Final Biomass and Chlorophyll Content of the Basal and Apical Ramets

**Figure 1:**
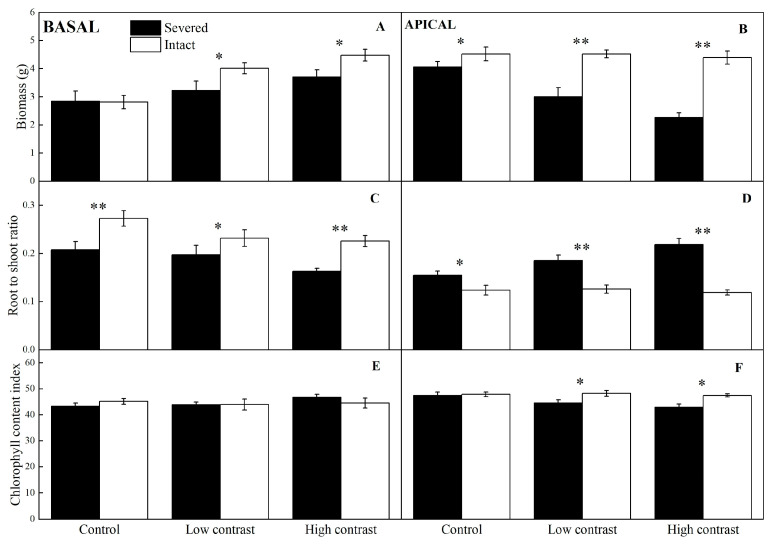
Effects of experimental treatments on the final biomass, root-to-shoot ratio, and relative chlorophyll content of basal (**A**,**C**,**E**) and apical (**B**,**D**,**F**) ramets of *A*. *philoxeroides*. The data indicate the means ± SE. * indicates *p* < 0.05 and ** indicates *p* < 0.01.

**Figure 2:**
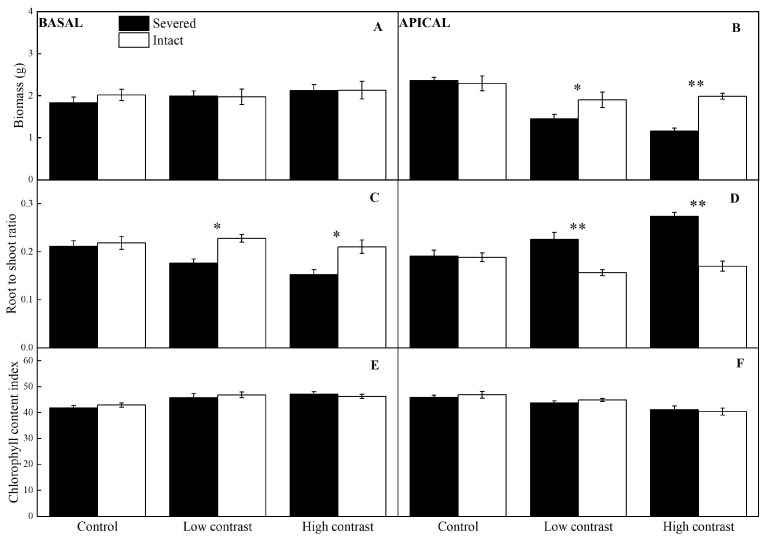
Effects of experimental treatments on the final biomass, root-to-shoot ratio, and relative chlorophyll content of basal (**A**,**C**,**E**) and apical (**B**,**D**,**F**) ramets of *A*. *sessilis*. The data indicate the means ± SE. * indicates *p* < 0.05 and ** indicates *p* < 0.01.

Clonal integration significantly increased the biomass of the apical ramets of *A*. *philoxeroides* and *A*. *sessilis*, and the positive effects of clonal integration were always significantly greater in *A*. *philoxeroides* than in *A*. *sessilis*, as indicated by significant species * integration effects (Table 1, Figure 1B and Figure 2B). Clonal integration significantly increased the chlorophyll content index of apical ramets in *A*. *philoxeroides* but not in *A*. *sessilis* under low and high contrast (significant species * integration interactions in Table 1, Figure 1F and Figure 2F). For the basal ramets, clonal integration significantly increased the biomass of *A*. *philoxeroides* under high and low contrast rather than *A*. *sessilis* (significant species * integration interactions in Table 1, Figure 1A and Figure 2A).

### 2.2. Root-to-Shoot Ratio of the Basal and Apical Ramets

Clonal integration significantly increased the proportion of biomass allocated to roots in basal ramets (Figure 1C and Figure 2C), whereas it decreased it in apical ramets (Figure 1D and Figure 2D), and the effect of clonal integration on the root-to-shoot ratio for apical ramets under greater contrast was more significant, as indicated by significant integration * contrast effects (Table 1; Figure 1C,D and Figure 2C,D).

### 2.3. Final Biomass of the Whole Clonal Fragment

**Figure 3:**
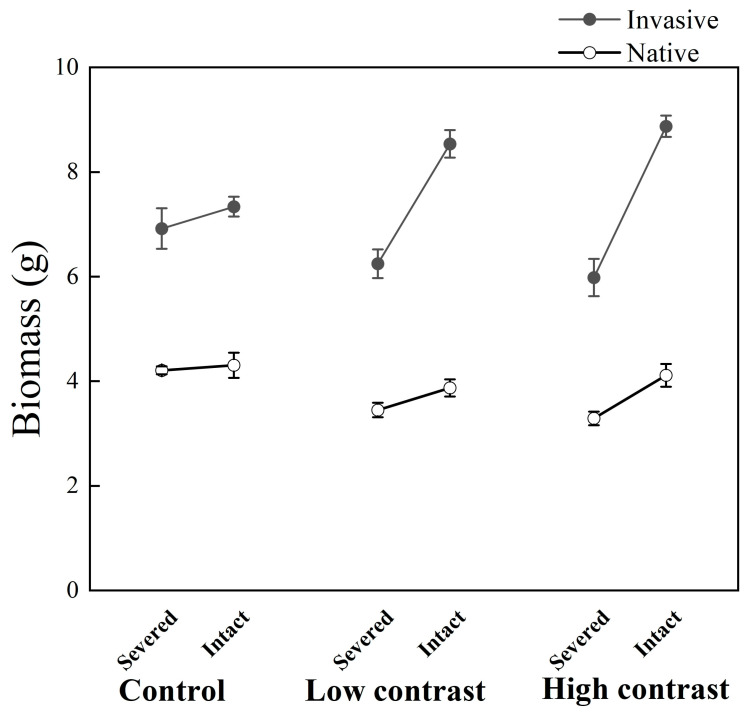
Biomass of the whole clone of *A*. *philoxeroides* and *A*. *sessilis* when the clonal fragment was grown under high-contrast, low-contrast, and control conditions with connections between the basal and apical ramets severed or kept intact. The data indicate the means ± SE.

Invasive *A*. *philoxeroides* accumulated more biomass than the native *A*. *sessilis* (Figure 3). Clonal integration significantly increased the final biomass of the whole clonal fragment, and the positive effects of clonal integration were always stronger under greater contrast, as indicated by significant integration * contrast effects (Table 1, Figure 3). These differences in the effect of the clonal integration among contrasts were greater in *A*. *philoxeroides* than in *A*. *sessilis*, as indicated by significant species * integration * contrast effects (Table 1, Figure 3).

## 3. Discussion

Theoretical studies predicted that greater patch contrast in a patchy habitat could lead to a stronger physiological integration [26,27]. Here we found the benefits of clonal integration increased with increasing patch contrast. More importantly, in support of our predictions, the results showed that the contribution of increased patch contrast to the benefits of clonal integration was greater in *A*. *philoxeroides* than in *A*. *sessilis*.

The ubiquity of environmental heterogeneity greatly increases the difficulty for plants to obtain and utilize essential resources, affects the growth and development of plants, and is associated with fitness [28,29]. In this study, without stolon connection, the biomass of the whole clonal fragments of *A*. *philoxeroides* and *A*. *sessilis* was significantly lower under high and low contrast than under the control treatments. However, when keeping the stolon connected, the growth performance of the plants under high and low contrast greatly benefited from clonal integration. Clonal integration may be an ecological adaptation strategy developed by clonal plants in response to environmental heterogeneity [30]. Many previous studies have also shown that ramets grown in low-resource patches can benefit from clonal integration and this can improve the growth performance and thus increase the fitness of the whole clone, due to the transfer of resources from connected donor ramets grown in high-resource patches [3,31]. Our study showed that this positive effect was more pronounced under greater patch contrast; this may be caused by more resource transfers due to stronger source–sink relationships. Irrespective of the effect of clonal integration, we found *A*. *philoxeroides* always had better growth and physiological performance in terms of biomass and relative chlorophyll content. This may be due to the stronger resource absorption and utilization capabilities of *A*. *philoxeroides* [32]. Rapid biomass production can provide *A*. *philoxeroides* with the fitness advantage needed to invade native communities.

We also found that clonal integration significantly increased the root-to-shoot ratio in the basal ramets, but significantly decreased it in the apical ramets. The reduced root-to-shoot ratio in the apical ramets allows them to focus on obtaining sufficient light and carbon dioxide, and the increased root-to-shoot ratio in the basal ramets allows them to focus on obtaining abundant soil nutrients. Because resource uptake is more economical in the patches where the resource is more abundant, this division of labor significantly increased the biomass of the whole clonal fragment [33]. Such division of labor is also common in previous studies [34,35], and may be one of the important ways for clonal integration to improve the performance of clonal plants [36,37].

No significant differences in the benefits of clonal integration of the whole clonal fragment between *A*. *philoxeroides* and *A*. *sessilis* were found under the control treatments. However, in an environment without patch contrast, clonal integration significantly increased the biomass of apical ramets of *A*. *philoxeroides* and did not significantly reduce the growth of basal ramets. This is consistent with the study by Xi et al. (2019). One possible explanation is that when the apical ramets are limited by their own absorptive capacity rather than the availability of external resources, they will benefit from physiological integration even in a homogeneous environment. Additionally, if the availability of external resources is high, the performance of the exported ramets will decrease less [13,38]. Clonal integration had no effect on the growth of *A*. *sessilis* under the control treatments. Although the effects of clonal integration on the whole clonal fragments between *A*. *philoxeroides* and *A*. *sessilis* did not show significant differences, considering the important role of young ramets in exploring new open spaces and rapid expansion [18,39], clonal integration may still confer *A*. *philoxeroides* an advantage over *A*. *sessilis* in some homogeneous habitats. 

Under two soil nutrient contrast treatments, clonal integration significantly increased the relative chlorophyll content of the apical ramets of *A*. *philoxeroides*, and thus increased the biomass of the whole clonal fragments of *A*. *philoxeroides* more than *A*. *sessilis.* Unexpectedly, clonal integration also significantly increased the growth performance of the basal ramets of *A*. *philoxeroides*. Possibly, the increased growth of basal ramets is a side effect of increased resource absorption and photosynthesis, which is triggered by the strong sink activity of the connected recipient ramets [40,41]. Clonal integration did not affect the biomass of the basal ramets of *A*. *sessilis*. This is consistent with many previous studies; one possible reason is that only surplus resources are transmitted by the donor ramets [42,43]. The obtained results demonstrated that *A. philoxeroides* had a stronger ability for clonal integration than *A. sessilis*, especially under higher patch contrast. 

Contrary to what we expected, the benefits of clonal integration on the whole clonal fragment of *A*. *philoxeroides* and *A*. *sessilis* were not significantly different between high and low contrast. We may have underestimated the ability of clonal integration of *A*. *philoxeroides* under low contrast. Since the high contrast we used represents some extreme cases that are not common at such fine scales, this discovery will have strong practical significance. Because it represents a heterogeneous nutrient habitat that is widespread in nature, clonal integration still confers a strong absolute growth advantage on *A*. *philoxeroides* over *A*. *sessilis*.

In terms of the absolute increase in biomass, we found that the invasive species *A*. *philoxeroides* benefited more from clonal integration than the native species *A*. *sessilis*. This may be because the basal ramets of *A*. *philoxeroides* could take up and use resources such as nutrients more efficiently than those of *A*. *sessilis* so a stronger source of nutrients could be created in basal ramets of *A*. *philoxeroides* than in *A*. *sessilis* [5]. This results in more resources being passed from the basal ramets to the apical ramets. Since absolute changes in biomass rather than relative changes are critical to competition, the differences in the ability of clonal integration may give *A*. *philoxeroides* an advantage over *A*. *sessilis* [5]. More importantly, our study shows that an increase in patch contrast can amplify this ability gap. Therefore, we can reasonably speculate that increasing the spatial heterogeneity of resource supply can facilitate the invasion of *A*. *philoxeroides*. This partly explains why the disturbed communities are often severely invaded by exotic plants [44,45,46].

To sum up, using a model clonal plant, *A*. *philoxeroides*, and its native co-genus, *A*. *sessilis,* as an experimental example, we found that clonal integration may play a more important role in the invasion process of invasive clonal plants in an environment with a greater degree of resource distribution heterogeneity. It is not only reflected in the promotion of the growth of foreign invaders but also in changing the growth contrast between the invasive clonal plant *A*. *philoxeroides* and its coexisting native plant *A*. *sessilis*. We suggest that further research should consider competition experiments between invasive clonal species and native clonal species, using multiple pairs of invasive and native species to explore the role and mechanism of clonal integration in the process of alien invasive species invading local communities under different resource patch contrasts.

## 4. Materials and Methods

### 4.1. Study Species and Plant Material

*Alternanthera philoxeroides* (Mart.) Griseb. is a creeping perennial herb of the Amaranthaceae family, native to South America [47]. *Alternanthera philoxeroides* is very invasive because of its high rate of spread and adaptability to a variety of environmental conditions. In its invasive area, the grass naturally sprawls and expands rapidly, encroaching on arable land and silting up rivers, seriously threatening the ecological environment of more than thirty countries including China, Australia, India, Laos, Thailand, Mexico, and the United States [48]. In the last decade, this species has been frequently used as a model clonal plant species to examine ecological questions [49]. In China, *A*. *philoxeroides* is an asexually reproductive plant with low genetic diversity, and studies have found that even different populations still have the same genotype [50].

*Alternanthera sessilis* (L.) DC. is a perennial herb with two reproductive modes: sexual reproduction and clonal reproduction. *A*. *sessilis* is mainly distributed in moist habitats such as swamps, wetlands, and the edges of ditches and canals [51]. *Alternanthera philoxeroides* and *A*. *sessilis* have similar morphological characteristics. In natural environments, these two plant species often co-exist in diverse habitats from wet to aquatic in south China.

In April 2021, we collected the plant material of *A*. *philoxeroides* and *A*. *sessilis* from the surrounding wetlands of Gonghu Bay, Taihu Lake, Jiangsu Province, China (N 31°25′–31°28′, E 120°15′–120°21′). To enhance the probability of collecting plant material from different genotypes (genes), we obtained multiple fragments of each species from various locations separated by over 500 m. Then, plants from different locations were mixed and propagated in the greenhouse. During the experimental period, the two plant species were watered and sprayed with acaricide to ensure that the two plants were not disturbed by too many external factors before the experiment.

### 4.2. Experimental Design

**Figure 4:**
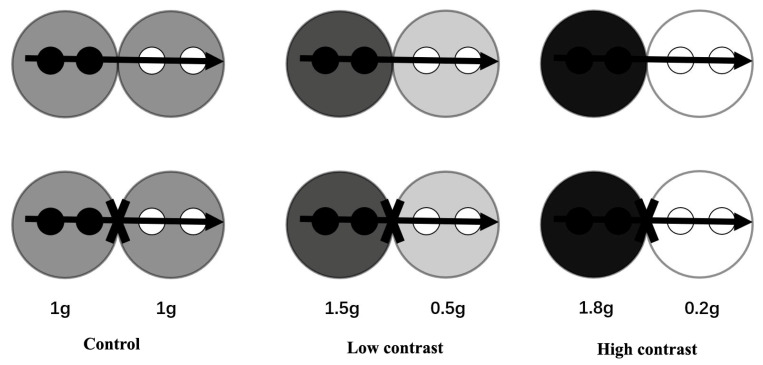
Schematic representation of the experimental design. There were 12 experimental treatments involving species, stolon connection (clonal integration), and patch contrast. The clonal fragments of *A. philoxeroides* and *A. sessilis*, each consisting of two basal ramets (solid circles) and two apical ramets (hollow circles) with a stolon apex (horizontal arrow), were grown under control, low contrast, and high contrast conditions, respectively, and with the stolon connections between basal and apical ramets either intact or severed.

The experiments were conducted in a greenhouse on the campus of Jiangsu University, Zhenjiang, China. In mid-August 2021, 30 similar-sized clonal fragments (tip cuttings, 15.23 ± 0.21 cm in length, 0.53 ± 0.034 g in dry mass for *A*. *philoxeroides*; 16.61 ± 0.18 cm in length, 0.74 ± 0.047 g in dry mass for *A*. *sessilis*) were selected for the experiment for each species. No differences were detected in initial size for both plant species (*p* > 0.05, One-way ANOVA). Each clonal fragment contained 4 ramets, of which, the two older ramets were called the basal part (close to the mother ramets), and the younger two ramets were called the apical part (distal to the mother ramets). The container used in the experiment was a rectangular plastic container with a length of 32 cm, a width of 18 cm, and a height of 8 cm. The container was divided into two parts with a fixed partition in the middle that isolated the water, nutrients, and other substances. For each clonal fragment, two ramets of the basal part were placed within basal section of a container and the other two ramets and apex of the apical part were within the apical section of the same container. The stolon of the apical ramets was anchored to the soil surface to facilitating rooting (see Figure 4). The substrate was a mixture of washed river sand and green zeolite (water retention) in a volume ratio of 3:1.

After a week of plant recovery, we began the experiments with a factorial design to test the effects of species (*A*. *philoxeroides* vs. *A*. *sessilis*), clonal integration (stolon connection represents clonal integration allowed, cutting off stolon connection represents prevention of clonal integration), and nutrient contrast. Contrast included three treatments: high contrast, low contrast, and control (no contrast). High contrast treatment was achieved by adding 1.8 g of slow-release fertilizer (Osmocote^R^, N–P–K: 16–9–12) to the basal part of the plastic container and 0.2 g to the apical part. The values of low contrast and control treatment were 1.5, 0.5 and 1, 1 respectively (see Figure 4). There were 12 treatments in the experiment, and each treatment had 5 replicates.

During the experimental period, the mean light intensity was 1200–1500 µmol m^−2^s^−1^ at noon and the mean air temperature was 28–35 °C in the greenhouse. The experimental units were randomly repositioned every two weeks to avoid the effects of possible environmental heterogeneity (such as light), and regular watering was used to meet the needs of plant growth. After about 8 weeks, the experiment ended in mid-October 2021.

### 4.3. Measurements

Relative chlorophyll content was measured on a clear morning in direct sunlight three days before plant harvest. A pair of well-developed leaves were selected from the apical and basal ramets of each plant fragment, and the relative chlorophyll content was measured using a portable chlorophyll meter (TYS-A, TOP, Zhejiang, China). Each leaf in a pair of leaves was measured once, and after the operation error was eliminated, the average value of the two values was taken as the measured value. At the harvest, all the ramets were divided into the apical and the basal part. Then, the roots, stems, and leaves were harvested, numbered, and baked at a constant temperature of 80 °C for 72 h, and weighed separately. 

### 4.4. Statistical Analysis

We used a generalized linear model to test the effects of species (*A*. *philoxeroides* vs. *A*. *sessilis*), integration (severed vs. intact), contrast (control vs. low vs. high), and their interactions on the final biomass, root-to-shoot ratio, chlorophyll content index of the apical and basal ramets, and the biomass of the whole clonal fragment. Post hoc least significance difference tests were used to determine if connection changed the response variables for each species under each contrast treatment. Statistical significance was assigned at *p* < 0.05. All data analyses were performed by SPSS 26 (SPSS, Chicago, IL, USA).

## Figures and Tables

**Table 1:** Results of generalized linear models for effects of contrast, species, and integration on the final biomass of the whole clone, and the final biomass, root-to-shoot ratio, and relative chlorophyll content of the basal and apical ramets.

Dependent Variable	Contrast (C)		Integration (I)		Species (S)		C × I		C × S		I × S		C × I × S	
	χ^2^	P	χ^2^	P	χ^2^	P	χ^2^	P	χ^2^	P	χ^2^	P	χ^2^	P
**Apical**														
Final biomass	61.552	**<0.001**	92.260	**<0.001**	428.52	**<0.001**	33.513	**<0.001**	2.208	0.332	27.759	**<0.001**	3.079	0.215
R/S ratio	25.315	**<0.001**	140.267	**<0.001**	81.147	**<0.001**	45.557	**<0.001**	2.142	0.343	0.196	0.658	2.805	0.246
Chlorophyll content index	36.482	**<0.001**	8.472	**0.004**	22.152	**<0.001**	1.632	0.442	5.871	0.053	4.945	**0.026**	4.529	0.104
**Basal**														
Final biomass	27.881	**<0.001**	5.966	**0.015**	174.54	**<0.001**	1.637	0.441	15.251	**<0.001**	3.970	**0.046**	4.248	0.120
R/S ratio	22.196	**<0.001**	45.128	**<0.001**	5.840	**0.016**	2.201	0.333	0.779	0.677	1.315	0.252	5.169	0.075
Chlorophyll content index	12.941	**0.002**	0.030	0.863	0.581	0.446	3.673	0.159	7.039	**0.030**	0.157	0.692	0.558	0.757
**Whole fragment**														
Final biomass	1.352	0.509	89.667	**<0.001**	792.98	**<0.001**	29.861	**<0.001**	10.943	**0.004**	33.655	**<0.001**	10.183	**0.006**
df	2.48		1.48		1.48		2.48		2.48		1.48		2.48	

Values are in bold indicate *p* < 0.05.

## Data Availability

Data are available by request to the corresponding author.

## References

[1] Richardson D.M., Pysek P., Rejmánek M., Barbour M.G., Panetta F.D., West C.J. (2000). Naturalization and Invasion of Alien Plants: Concepts and Definitions. Divers. Distrib..

[2] Van Kleunen M., Dawson W., Essl F., Pergl J., Winter M., Weber E., Kreft H., Weigelt P., Kartesz J., Nishino M. (2015). Global exchange and accumulation of non-native plants. Nature.

[3] Wang J.N., Wang Q.Y., Hu J.W., Yu H.W., Liu C.H., Yu D. (2021). The influence of small-scale resource heterogeneity caused by human activities on the growth phenotype of invasive aquatic plants. Ecol. Indic..

[4] Song Y.B., Yu F.H., Keser L.H., Dawson W., Fischer M., Dong M., van Kleunen M. (2013). United we stand, divided we fall: A meta-analysis of experiments on clonal integration and its relationship to invasiveness. Oecologia.

[5] Wang Y.J., Müller-Schärer H., van Kleunen M., Cai A.M., Zhang P., Yan R., Dong B.C., Yu F.H. (2017). Invasive alien plants benefit more from clonal integration in heterogeneous environments than natives. New Phytol..

[6] Keser L.H., Dawson W., Song Y.B., Yu F.H., Fischer M., Dong M., Kleunen M. (2014). Invasive clonal plant species have a greater root-foraging plasticity than non-invasive ones. Oecologia.

[7] Liu J., Dong M., Miao S.L., Li Z.Y., Song M.H., Wang R.Q. (2006). Invasive alien plants in China: Role of clonality and geographical origin. Biol. Invasions.

[8] You W.H., Yu D., Liu C.H., Xie D., Xiong W. (2013). Clonal integration facilitates invasiveness of the alien aquatic plant *Myriophyllum aquaticum* L. under heterogeneous water availability. Hydrobiologia.

[9] Chen D., Ali A., Yong X.H., Lin C.G., Niu X.H., Cai A.M., Dong B.C., Zhou Z.X., Wang Y.J., Yu F.H. (2019). A multi-species comparison of selective placement patterns of ramets in invasive alien and native clonal plants to light, soil nutrient and water heterogeneity. Sci. Total Environ..

[10] de Kroon H., van der Zalm E., van Rheenen J.W., van Dijk A., Kreulen R. (1998). The interaction between water and nitrogen translocation in a rhizomatous sedge (*Carex flacca*). Oecologia.

[11] Wang N., Yu F.H., Li P.X., He W.M., Liu F.H., Liu J.M., Dong M. (2008). Clonal integration affects growth, photosynthetic efficiency and biomass allocation, but not the competitive ability, of the alien invasive *Alternanthera philoxeroides* under severe stress. Ann. Bot..

[12] Caldwell M.M. (1994). Exploitation of Environmental Heterogeneity by Plants: Ecophysiological Processes Above- and Belowground. Ecology.

[13] Xi D.G., You W.H., Hu A.A., Huang P., Du D.L. (2019). Developmentally Programmed Division of Labor in the Aquatic Invader *Alternanthera philoxeroides* Under Homogeneous Soil Nutrients. Front. Plant Sci..

[14] Hutchings M.J., Turkington R., Klein E., Peter C. (2011). Morphological plasticity in *Trifolium repens* L.: The effects of clone. Can. J. Bot..

[15] Stuefer J.F., Hutchings M.J. (1994). Environmental heterogeneity and clonal growth: A study of the capacity for reciprocal translocation in *Glechoma hederacea* L.. Oecologia.

[16] Roiloa S.R., Rodriguez-Echeverria S., de la Pena E., Freitas H. (2010). Physiological integration increases the survival and growth of the clonal invader *Carpobrotus edulis*. Biol. Invasions.

[17] Alpert P. (1999). Clonal integration in *Fragaria chiloensis* differs between populations: Ramets from grassland are selfish. Oecologia.

[18] You W.H., Fan S.F., Yu D., Xie D., Liu C.H. (2014). An invasive clonal plant benefits from clonal integration more than a co-occurring native plant in nutrient-patchy and competitive environments. PLoS ONE.

[19] Yu F.H., Wang N., Alpert P., He W.M., Dong M. (2009). Physiological integration in an introduced, invasive plant increases its spread into experimental communities and modifies their structure. Am. J. Bot..

[20] Stuefer J.F. (1996). Potential and Limitations of Current Concepts regarding the Response of Clonal Plants to Environmental Heterogeneity. Vegetatio.

[21] Kotliar N.B., Wiens J.A. (1990). Multiple Scales of Patchiness and Patch Structure: A Hierarchical Framework for the Study of Heterogeneity. Oikos.

[22] Wei G., Song Y.B., Yu F.H. (2018). Heterogeneous light supply affects growth and biomass allocation of the understory fern *Diplopterygium glaucum* at high patch contrast. PLoS ONE.

[23] Lin H.F., Alpert P., Zhang Q., Yu F.H. (2018). Facilitation of amphibious habit by physiological integration in the clonal, perennial, climbing herb *Ipomoea aquatica*. Sci. Total Environ..

[24] Li Y.H., Wang Z.W., Ma H.L. (2008). Patchy contrast of habitat affects intraclonal division of labor of *Potentilla anserina*. J. Plant Ecol..

[25] Zhang L.L., He W.M. (2008). Consequences of ramets helping ramets: No damage and increased nutrient use efficiency in nurse ramets of *Glechoma longituba*. Flora.

[26] Caraco T., Kelly C.K. (1991). On the adaptive value of physiological integration in clonal plants. Ecology.

[27] Wang Z.W., Li Y.H., During H.J., Li L.H. (2011). Do Clonal Plants Show Greater Division of Labour Morphologically and Physiologically at Higher Patch Contrasts?. PLoS ONE.

[28] Dong M. (1995). Morphological responses to local light conditions in clonal herbs from contrasting habitats, and their modification due to physiological integration. Oecologia.

[29] Yu F.H., Chen Y.F., Dong M. (2001). Clonal integration enhances survival and performance of *Potentilla anserina*, suffering from partial sand burial on Ordos plateau, China. Evol. Ecol..

[30] Liu F.H., Liu J., Dong M. (2016). Ecological Consequences of Clonal Integration in Plants. Front. Plant Sci..

[31] Si C., Xue W., Guo Z.W., Zhang J.F., Hong M.M., Wang Y.Y., Lin J., Yu F.H. (2021). Soil heterogeneity and earthworms independently promote growth of two bamboo species. Ecol. Indic..

[32] Zhao C.Y., Liu Y.Y., Shi X.P., Wang Y.J. (2020). Effects of soil nutrient variability and competitor identify on growth and co-existence among invasive alien and native clonal plants. Environ. Pollut..

[33] Huang Q.Q., Li X.X., Huang F.F., Wang R.L., Lu B.Q., Shen Y.D., Fan Z.W., Lin P.Q. (2018). Nutrient addition increases the capacity for division of labor and the benefits of clonal integration in an invasive plant. Sci. Total Environ..

[34] Wang Y.J., Shi X.P., Meng X.F., Wu X.J., Luo F.L., Yu F.H. (2016). Effects of Spatial Patch Arrangement and Scale of Covarying Resources on Growth and Intraspecific Competition of a Clonal Plant. Front. Plant Sci..

[35] Zhang Y.C., Zhang Q.Y. (2013). Clonal Integration of *Fragaria orientalis* in Reciprocal and Coincident Patchiness Resources: Cost-Benefit Analysis. PLoS ONE.

[36] Friedman D., Alpert P. (1991). Reciprocal transport between ramets increases growth of *Fragaria chiloensis* when light and nitrogen occur in separate patches but only if patches are rich. Oecologia.

[37] Stuefer J. (1998). Two types of division of labour in clonal plants: Benefits, costs and constraints. Perspect. Plant Ecol. Evol. Syst..

[38] Dong B.C., Alpert P., Zhang Q., Yu F.H. (2015). Clonal integration in homogeneous environments increases performance of *Alternanthera philoxeroides*. Oecologia.

[39] Li X.X., Fan Z.W., Shen Y.D., Wang W. (2019). Nutrient addition does not increase the benefits of clonal integration in an invasive plant spreading from open patches into plant communities. Plant Biol..

[40] Hartnett D.C., Bazzaz F.A. (1983). Physiological Integration among Intraclonal Ramets in Solidago Canadensis. Ecology.

[41] Zhang Y.C., Zhang Q.Y., Peng L., Wu N. (2009). Photosynthetic response of *Fragaria orientalis* in different water contrast clonal integration. Ecol. Res..

[42] Chen J.S., Li J., Yun Z., Hao Z. (2015). Clonal integration ameliorates the carbon accumulation capacity of a stoloniferous herb, *Glechoma longituba*, growing in heterogenous light conditions by facilitating nitrogen assimilation in the rhizosphere. Ann. Bot..

[43] Stuefer J.F., During H.J., Kroon H. (1994). High benefits of clonal integration in two stoloniferous species, in response to heterogenous. J. Ecol..

[44] Bradley B.A., Blumenthal D.M., Wilcove D.S., Ziska L.H. (2010). Predicting plant invasions in an era of global change. Trends Ecol. Evol..

[45] Kalwij J.M., Robertson M.P., Rensburg B. (2015). Annual monitoring reveals rapid upward movement of exotic plants in a montane ecosystem. Biol. Invasions.

[46] Lembrechts J.J., Pauchard A., Lenoir J., Nuñez M.A., Geron C., Ven A., Bravo-Monasterio P., Teneb E., Nijs I., Milbau A. (2016). Disturbance is the key to plant invasions in cold environments. Proc. Natl. Acad. Sci. USA.

[47] Schmid R., Holm L.R., Doll J., Holm E., Herberger J. (1997). World weeds: Natural histories and distribution. Weed Technol..

[48] Wang N., Yu F.H., Li P.X., He W.M., Liu J., Yu G.L., Song Y.B., Dong M. (2009). Clonal integration supports the expansion from terrestrial to aquatic environments of the amphibious stoloniferous herb *Alternanthera philoxeroides*. Plant Biol..

[49] Dong B.C., Meng J., Yu F.H. (2019). Effects of parental light environment on growth and morphological responses of clonal offspring. Plant Biol..

[50] Wang B.R., Li W.G., Wang J.B. (2005). Genetic diversity of *Alternanthera philoxeroides* in China. Aquat. Bot..

[51] Wang J.T., Miao L.L., Liu C.H. (2016). The invasive stoloniferous clonal plant *Alternanthera philoxeroides* outperforms its co-occurring non-invasive functional counterparts in heterogeneous soil environments—Invasion implications. Sci. Rep..

